# Inflammation and tissue repair markers distinguish the nodular sclerosis and mixed cellularity subtypes of classical Hodgkin's lymphoma

**DOI:** 10.1038/sj.bjc.6605238

**Published:** 2009-09-22

**Authors:** A Birgersdotter, K R N Baumforth, A Porwit, J Sjöberg, W Wei, M Björkholm, P G Murray, I Ernberg

**Affiliations:** 1 Department of Microbiology, Tumor Biology and Cell Biology, Karolinska Institutet, Stockholm SE-171 77, Sweden; 2 School of Cancer Sciences, Cancer Research UK Institute for Cancer Studies, University of Birmingham, Birmingham, UK; 3 Department of Pathology, Karolinska University Hospital Solna and Karolinska Institutet, Stockholm SE-171 77, Sweden; 4 Division of Hematology, Department of Medicine, Karolinska University Hospital Solna and Karolinska Institutet, Stockholm SE-171 77, Sweden

**Keywords:** Hodgkin's lymphoma, gene expression, wound healing, microenvironment

## Abstract

**Background:**

Classical Hodgkin's lymphoma (cHL), although a malignant disease, has many features in common with an inflammatory condition. The aim of this study was to establish the molecular characteristics of the two most common cHL subtypes, nodular sclerosis (NS) and mixed cellularity (MC), based on molecular profiling and immunohistochemistry, with special reference to the inflammatory microenvironment.

**Methods:**

We analysed 44 gene expression profiles of cHL whole tumour tissues, 25 cases of NS and 19 cases of MC, using Affymetrix chip technology and immunohistochemistry.

**Results:**

In the NS subtype, 152 genes showed a significantly higher expression, including genes involved in extracellular matrix (ECM) remodelling and ECM deposition similar to wound healing. Among these were *SPARC*, *CTSK* and *COLI*. Immunohistochemistry revealed that the NS-related genes were mainly expressed by macrophages and fibroblasts. Fifty-three genes had a higher expression in the MC subtype, including several inflammation-related genes, such as *C1Qα*, *C1Qβ* and *CXCL9*. In MC tissues, the C1Q subunits were mainly expressed by infiltrating macrophages.

**Conclusions and interpretations:**

We suggest that the identified subtype-specific genes could reflect different phases of wound healing. Our study underlines the potential function of infiltrating macrophages in shaping the cHL tumour microenvironment.

Classical Hodgkin's lymphoma (cHL), although a malignant disease, has many features in common with an inflammatory condition. cHL often presents with symptoms of fever, night sweats, itching, lymphadenopathy and splenomegaly. Laboratory findings include neutrophilia, eosinophilia, lymphocytopenia and altered serum phase reactants. Classical Hodgkin's lymphoma is characterised by the presence of Hodgkin's Reed–Sternberg (H-RS) cells. The H-RS cells originate from transformed pre-apoptotic germinal centre B-cells that have lost their capacity to express a high-affinity B-cell receptor (Kuppers *et al*, 1994; Brauninger *et al*, 2006). The H-RS cells constitute only a small minority (∼1%) of the cell population of the tumour. The inflammatory cellular infiltrate in the cHL tumour tissue is heterogeneous, consisting of lymphocytes, macrophages, eosinophils, mast cells, plasma cells and fibroblasts. There is evidence that these infiltrating cells are involved in a reactive inflammatory process creating an environment that allows and probably promotes the survival of H-RS cells (Poppema and van den Berg, 2000; Aldinucci *et al*, 2002). In cHL, cytokines and chemokines operate in a complex interaction, which seems to be important for the pathogenesis of cHL (Re *et al*, 2005). Several studies indicate that the release of biologically active mediators from H-RS cells, such as cytokines (e.g., interleukin (IL) 13), has an important function in the pathophysiology of cHL (Skinnider *et al*, 2001; Trieu *et al*, 2004).

On the basis of morphology, cHL is divided into four subtypes: nodular sclerosis (NS), mixed cellularity (MC), lymphocyte rich and lymphocyte depleted. Ninety percent of cHL cases belong to the MC or NS subtype. The composition of the tumour tissue differs between the NS and MC subtypes. Nodular sclerosis subtype is usually associated with eosinophilia and high numbers of CD4-positive T cells. Nodular sclerosis also has a characteristic desmoplasia and displays a distinct extracellular matrix (ECM) deposition. Mixed cellularity is often characterised by the presence of epithelioid macrophages. Earlier gene expression studies of whole-tissue cHL tumour biopsies have identified possible prognostic markers as well as genes affected by the Epstein–Barr Virus (EBV) (Devilard *et al*, 2002; Sanchez-Aguilera *et al*, 2006; Baumforth *et al*, 2008).

The primary aim of this study was to establish the molecular characteristics of the two major cHL subtypes, NS and MC, using gene expression profiling and immunohistochemistry.

To visualise the cell type(s) expressing the subtype-specific genes, the same tumours were analysed by immunohistochemistry. Our results indicate that macrophages have an important function in shaping the tumour microenvironment in cHL.

## Materials and methods

### Patients

Diagnostic lymph node biopsies from cHL patients were collected from 1994 to 2004 at the Department of Pathology and Cytology, Karolinska University Hospital Solna, Sweden (*n*=32) and obtained from the Children's Cancer and Leukaemia Group (CCLG), UK (*n*=10). Of the Swedish samples, one NS and one MC tumour was arrayed twice, making the number of tumour gene expression profiles 44. The median age of all patients was 20 years (range; 6–90); the median age of NS patients was 17 (range; 13–69), and it was 52 for MC patients (range; 6–90). The male/female ratio was 16 : 6 in the NS group and 14 : 5 in the MC group. Of 41 of 42 tumours analysed for EBV status, 17 were EBV-positive. These included 7 of 24 analysed NS tumours and 10 of 18 analysed MC tumours.

This study was performed with ethics approval from the South Birmingham Research Ethics Committee (LREC no. 0844) and from the Karolinska Institutet Research Ethics Committee North (approval number 01-004).

### Samples

Fragments of fresh lymph node biopsies were snap-frozen in liquid nitrogen and stored at −80°C. The cHL cases were diagnosed according to the criteria of the WHO classification (Jaffe *et al*, 2001). Routine morphological and immunohistochemical staining (CD30, CD20, CD3, CD15, ALK-1), which were necessary to establish diagnosis, were carried out on paraffin sections. EBV expression was investigated by immunohistochemistry (latent membrane protein 1) and *in situ* hybridisation (EBV encoded RNA) as described earlier (Axdorph *et al*, 1999).

### Morphological evaluation

A comprehensive evaluation of the morphology and infiltration by various cell types was performed on 28 of 42 tumours by one of us (AP). The degree of eosinophilia and the number of H-RS cells were determined by the random selection of 10 consecutive high power fields (HPFs) in haematoxylin–eosin-stained paraffin sections. In each HPF, the number of eosinophils or H-RS cells was determined and the sum for 10 HPF was calculated. The biopsies were then classified as low eosinophilia (<50 eosinophils/10 HPF), medium eosinophilia (50–120 eosinophils/10 HPF) or high eosinophilia (>120 eosinophils/10 HPF). The number of H-RS cells was detected by CD30 immunohistochemistry and classified as few (<5 H-RS cells/10 HPFs), medium (5–10 H-RS cells/10 HPFs) or many (>10 H-RS cells/10 HPFs).

The numbers of infiltrating macrophages were evaluated on the basis of CD68 staining and/or morphology and the tumours were divided into two groups: with high and low macrophage counts.

The degree of fibrosis was classified into four groups based on morphology and reticulin staining (Gordon-Sweet); 0 for no fibrosis, 1 for slight fibrosis, 2 for advanced fibrosis, 3 for highly advanced fibrosis (Table 1).

### RNA preparation and evaluation

RNA extraction from biopsies was performed either with the Ultraspec-II RNA isolation system (Biotecx, Houston, TX, USA) according to the manufacturer's protocol or with the Qiagen RNeasy minikit (Valencia, CA, USA) with a minor modification, that is, the amount of lysis buffer was increased to 500 *μ*l.

### Microarrays

The labelling and hybridisation were performed according to the standard Affymetrix protocols at the KI Microarray Core Facility, Novum, Huddinge, Sweden (http://www.bea.ki.se/affymetrix/) or at Cancer Research UK (CRUK), Birmingham University, Birmingham, UK. Two different array Affymetrix designs were used: the U133+2 and the human genome focus (HGF) arrays containing 8793 genes (including internal controls). The probe sets in the HGF arrays are a subset of those present on the U133+2 arrays, which makes them comparable.

### Bioinformatics

We used the Affymetrix GCOS (Santa Clara, CA, USA) software to convert the CEL files into absolute statistical signals and the samples were scaled to the average signal of 100. The first step in the analysis was to assess the quality reports. Our inclusion criteria were as follows: average present call above 40% for all the genes, background <90, background s.d. <2, scaling factor <2 and degradation of GAPDH as measured by 3′/5′-end probes ratio <2. All samples passed four out of five criteria and were thus included in the further analysis.

The software used to analyse the gene expression patterns in NS and MC tumours were as follows: the Gene Expression Dynamics Inspector (GEDI), the Significant Analysis of Microarrays (SAM, http://www-stat.stanford.edu/~tibs/SAM/) and the dChip software (www.dChip.org). GEDI is a Matlab freeware program that uses self-organising maps (SOMs) to translate high-dimensional data into a 2D mosaic (http://www.childrenshospital.org/research/ingber/GEDI/GEDIhome.htm).

The Gene Expression Dynamics Inspector is population-based rather than gene-based, and is thus fit to analyse large amounts of data. Each tile of the mosaic represents an individual SOM cluster and is colour-coded to represent over- and under-expression of the genes in a cluster, thus identifying the underlying gene pattern (Eichler *et al*, 2003).

The CEL files were converted and normalised using the robust multichip average method and quintile normalisation before further analysis (Irizarry *et al*, 2003). The SAM software was used for the identification of differentially expressed genes (using 1.7 as a fold change cutoff, and 5% false discovery rate) (Tusher *et al*, 2001). We used SAM to investigate the correlation between gene expression and fibrosis or eosinophilia (for 28 of the samples), and dChip 1.3 was used for cluster analysis.

Fisher's exact test was applied for statistical analysis of the correlation between morphological/immunohistochemical studies and gene expression analysis.

### Real-time–PCR

For selected genes, the real-time quantitative (RT)–PCR was performed to validate the microarray results. The same RNA samples from 22 of the tumours were used as templates for c-DNA reaction in the Reverse Transcription System (Promega, Madison, WI, USA).

Taqman gene expression assays (Applied Biosystems, Foster City, CA, USA) were used with pre-made primers and FAM-MGB probes for secreted protein acidic and rich in cysteins *(SPARC)* (Hs00234160), *CCL22* (Hs0017080), *CXCL9* (Hs0017065), *CCL17* (Hs0017074) and *C1QB* (Hs00608019). Two different types of controls were included: *β*-actin was used as an internal control and EBV Namalwa DNA was used as a standard (Enbom *et al*, 2001).

### Validation of microarray results using immunohistochemistry

Immunohistochemistry was performed as described earlier using Dako's Envision kit (K5007, Dako, Glostrup, Denmark) (Reynolds *et al*, 2002). The following antibodies were used: SPARC (AON-5031, Haematologic Technologies Inc., Essex, Vermont, USA), osteoblast-specific factor 2 (OSF2) (ab14041, Abcam, Cambridge, UK), C1Q (A0136, Dako), CXCL9 (AF392, R&D Systems Inc., Abingdon, UK), AIM (3805, ProSci Inc., Poway, CA, USA) and cathepsin K (CTSK) (ab37259, Abcam).

Dilutions used in the experiments were as follows: SPARC 1 : 100, OSF2 1 : 100, C1Q 1 : 200, AIM 1 : 100 and CTSK 1 : 50.

Of the 44 tumours analysed earlier by microarrays, paraffin blocks from 28 samples were available for immunohistochemical analysis.

The results were scored without knowledge of the gene expression results and the samples were arbitrarily divided into groups according to the level of protein expression and/or the patterns of distribution of positive cells (Table 2).

## Results

### Gene expression profiling discriminated between cHL subtypes

**Figure 1:**
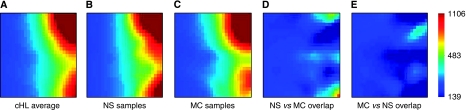
Gene expression profiles of cHL tumour samples visualised by the GEDI software. Genes with similar expression levels were grouped within the same tile; the red tiles represent highly expressed genes, yellow tiles intermediate expression and dark blue tiles represent genes with low expression. The colour bar on the lower right shows the signal intensities represented by different colours. Map (**A**) shows all included tumours, and maps (**B**) and (**C**) show samples divided according to the histopathological subtype. Maps (**D**) and (**E**) visualise the NS *vs* MC and MC *vs* NS comparisons. Map (**D**) shows a cluster of genes that have a higher expression in the NS subtype. However, a similar cluster is lacking in the (**E**) map.

Gene expression profiles of 25 NS and 19 MC cHL samples were visualised and compared using the GEDI software (Figure 1). Gene Expression Dynamics Inspector allows the comparison of profiles based on the direct visual detection of transcriptome patterns. Such intuitive ‘gestalt’ perception promotes the visualisation and discovery of interesting relationships. Figure 1B and C show that gene expression differs between the NS and MC subtypes of cHL. Figure 1D shows a cluster of active genes specific to NS tumours. When overlapping the MC and NS GEDI maps, no distinctive pattern could be seen besides the lack of NS ‘specific’ genes in MC samples (Figure 1E). Thus, the MC gene expression profile seems to be partly defined by the low expression of NS ‘specific’ genes. Alternatively, the gene expression patterns in MC cases might be more heterogeneous (i.e., the variation within the MC group of samples is higher than within the NS group).

**Figure 2:**
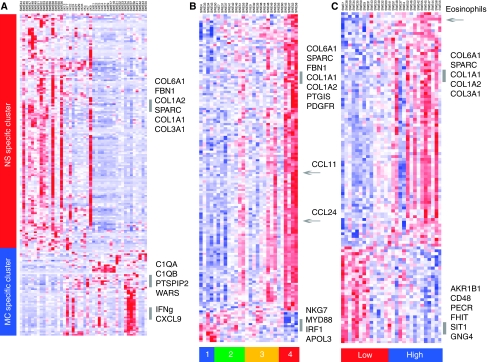
(**A**) Shows the clustering of the 205 genes that differed between the NS and the MC subtypes of cHL. Most of the genes that have a higher expression in the NS samples tended to be associated with the extracellular matrix, whereas the genes regulated by interferon had a higher expression in the MC samples. (**B**) The heat-map shows genes associated with fibrosis. The samples were divided according to the level of fibrosis: none and low *vs* high and extensive (i.e., 0 and 1 *vs* 2 and 3). (**C**) The heat-map shows the comparison between samples with high eosinophilia and low eosinophilia. Several of the genes that are associated with NS have a high expression in samples with high eosinophilia, whereas genes involved in Toll receptor signalling have a low expression. Arrows marking CCL11 and CCL24 are also known as eotaxin 1 and 2. The number of eosinophils is also included in the third down from the top heat-map, also marked by an arrow. The three heat-maps show that the NS-related genes were also associated with fibrosis and eosinophilia. However, the genes associated with MC are not included among the genes associated with low fibrosis or low eosinophilia.

The SAM software was used for the identification of differentially expressed genes (using 1.7 as a fold change cutoff, and 5% false discovery rate). Significant Analysis of Microarrays revealed that 152 genes had a higher relative expression in the NS cHL, whereas 53 genes had a higher expression in the MC samples. The high number of NS-related genes and the finding that MC is characterised by the lack of these genes fit well with the abovementioned results of the GEDI analysis. Selected differentially expressed genes are given in Table 3 and the list of all genes that differed is provided in Supplementary Table 1. It should be mentioned that genes coding for ECM and for proteins involved in ECM remodelling: collagen 1 (*COLI*), collagen III (*COLIII*), lumican (*LUM*), laminin-*β*1 (*LAMB1*), metallomatrixproteinase 2 (*MMP2*), *CTSK*, connective tissue growth factor (*CTGF*), *SPARC* and OSF2 were characteristic for the NS cHL (Table 3). Moreover, some genes involved in inflammation, including *CCL17*, *CCL22* and interleukin 9 (*IL9*) showed higher expression in NS samples. Several of these genes are associated with late inflammatory responses in wound healing. The genes characteristic for MC samples included inflammatory molecules: C1q subunits *C1Qα* and *C1Qβ*, *CXCL9*, *CD5L*, erythropoietin receptor and apolipoprotein C1. Other MC-related genes are known to be associated with cytotoxic T cells: *CD8*, *GRZA* and *GRZK*. Cluster analysis clearly showed a division between the two subgroups (Figure 2A).

The expression levels of five genes (*SPARC*, *CCL17*, *CCL22*, *CXCL9* and *C1Qβ*) were validated using real-time–PCR (RT–PCR), which showed a good correlation with the microarray expression values (Table 4).

### Comparison of gene expression, cellular infiltrate and degree of fibrosis

Twenty-eight of the tumours were further examined to establish the relationship between various gene expression patterns, the frequency of different infiltrating cell types and the degree of fibrosis (Table 1). Each histopathological variable was correlated with gene expression separately for each of the two subtypes. It has been reported earlier that eosinophilia and fibrosis are associated with the NS subtype, so we wanted to analyse whether the difference seen between MC and NS was purely due to a difference in fibrosis or a difference in tissue eosinophilia. Indeed, fibrosis was weakly associated and eosinophilia was associated with NS in our material (Fisher's exact test, *P-*value 0.057 and 0.054, respectively). There was no difference between the subtypes regarding the level of macrophage infiltration.

The level of fibrosis was denoted as low (0 for no fibrosis, 1 for slight fibrosis) or high (2 for advanced fibrosis and 3 for very advanced fibrosis) (Table 1). Comparison of samples with low and high fibrosis using the SAM software gave 144 genes with significantly different expression. The cluster analysis (Figure 2) showed that the NS-related genes were also associated with both fibrosis and tissue eosinophilia. Many of these genes were ECM related. However, the MC-related genes were not associated with low fibrosis or low tissue eosinophilia. Only one MC-related gene, *GZMK*, could be identified from the cluster analysis heat-map as associated with no fibrosis (Figure 2B). Comparison of samples with high and low eosinophilia (below 70 and above 70/10 HPF) using the SAM software yielded 116 differentially expressed genes. Of the 32 genes that were associated with low tissue eosinophilia, five did overlap with the MC-related genes: *IRF3*, *GZMK*, *PRF1*, *NKG7* and *APOL3*.

### Cell-type-specific expression

**Figure 3:**
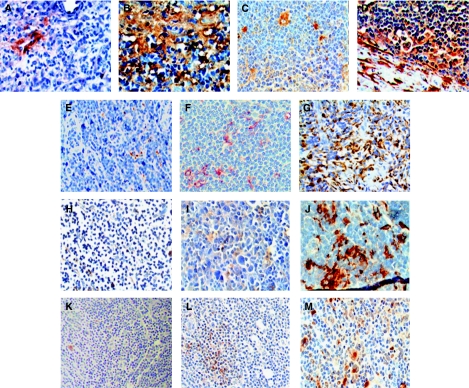
Cell-type-specific expression of selected gene products revealed by immunohistochemistry. The immunohistochemistry patterns summarised in Table 2 are illustrated. (**A**) Pattern I of OSF2 expression showing a positive staining around a vessel in an MC case. (**B**) Pattern II of OSF2 expression showing an extensive positive staining in fibrotic streaks surrounding nodules in an NS case. (**C**) Pattern I of SPARC expression showing positive staining in occasional macrophages in an MC case. (**D**) Pattern II of SPARC expression showing positive staining in macrophages and fibroblasts in an NS case. (**E**) Pattern I of CTSK expression showing occasional positive lymphocytes in an MC case. (**F**) Pattern II of CTSK expression with positive staining in macrophages and occasional lymphocytes in an MC case. (**G**) Pattern III of CTSK expression showing positive macrophages, occasional lymphocytes and fibroblasts in an NS case. (**H**) Pattern I of C1Q expression showing positive staining in a few lymphocytes and occasional macrophages in an NS case. (**I**) Pattern II of C1Q expression showing positive staining in macrophages in an NS case. (**J**) Pattern III of C1Q expression with many positive macrophages in an MC case. (**K**) Pattern I of CXCL9 expression with very scarce positive cells as shown in an NS case. (**L**) Pattern II of CXCL9 expression with small clusters of positive lymphocytes as shown in an NS case. (**M**) Pattern III of CXCL9 expression showing positive lymphocytes, macrophages and H-RS cells in an MC case.

To define which cell types were responsible for the high expression of the genes discriminating between the NS and MC cHL, the expression of five of the corresponding proteins was analysed by immunohistochemistry on paraffin sections (Figure 3, Tables 2 and 5).

The *OSF2* (a member of the Runt-related family of transcription factors that has a critical function during osteoblast differentiation) gene showed a 10-fold higher mRNA expression in the NS subtype when compared with the MC group, which ranked it as one of the most discriminating genes. The OSF2 protein was detected in fibrotic bands and in fibroblasts dispersed within these bands. The staining followed the fibrotic streaks of the tumours. The tumours were divided into two groups depending on the staining intensity (Table 2, Figure 3A and B). The number of NS tumours with high OSF2 expression (pattern II) was significantly higher than that of MC tumours (Table 5, Fisher's exact test, *P□*0.001).

Secreted protein acidic and rich in cysteine (SPARC), also known as osteonectin or BM-40, is a multifunctional glycoprotein that belongs to the matricellular group of proteins. It modulates cellular interaction with the ECM by its binding to structural matrix proteins, such as collagen and vitronectin, and by its abrogation of focal adhesions, which are features contributing to a counter-adhesive effect on cells. The SPARC antibody showed two distinct expression patterns (Table 2, Figure 3C and D), either showing positive staining in scattered macrophages (pattern I) or expression in both macrophages and fibroblasts (often within the sclerotic bands; pattern II). Occasionally, lymphocytes were positive. The expression pattern II was seen more often in NS cases (Table 5, Fisher's exact test, *P*=0.0047)

Cathepsin K, encoded by the *CTSK* gene, is a protease involved in bone resorption. Three staining patterns were found using the CTSK antibody (Table 2, Figure 3E, F and G). Pattern I showed low expression in a few scattered macrophages, pattern II had a strong expression seen mostly in macrophages and pattern III had the highest expression, which was seen in fibroblasts, macrophages and in a few lymphocytes. Pattern III was seen more often in NS samples (Table 5, Fisher's exact test *P*=0.0017). In some tumours, positive vessels and a few H-RS cells were found. There was variation in the staining patterns in both the MC and NS groups. In certain tumours, high numbers of positive macrophages were evenly distributed in the tissue. In other samples, the positive macrophages clustered around fibrotic bands or ECM deposits.

C1Q is a subcomponent of complement 1 (C1), which recognises and binds to the heavy chain of IgG or IgM, and initiates the classical complement pathway. The antibody against C1Q recognised both subunits-*α* and -*β*. Macrophages and lymphocytes were positive in most samples. There was no difference in the lymphocyte staining between the subtypes. Three C1Q staining patterns could be distinguished, depending on the numbers of stained macrophages (Table 2, Figure 3H, J and K). Vessels were also positive in a fraction of tumours. The staining pattern III, with most stained macrophages, was significantly more frequent in the MC group (Table 5, Fisher's exact test *P*=0.0067). In some MC cases, virtually all macrophages were stained. These samples were obtained from the oldest patients in the study.

Chemokine (C-X-C motif) ligand 9 (CXCL9) is a small cytokine belonging to the CXC chemokine family that is also known as Monokine induced by γ-interferon. Antibody to the CXCL9 protein stained lymphocytes, macrophages and, in some cases, H-RS cells. Three patterns were found: occasional positive staining in lymphocytes (I), positive staining in lymphocytes only (II) and positive staining in lymphocytes and macrophages (III) (Table 2, Figure 3L, M and N). Pattern III was found mainly in the MC group (Table 5, Fisher's exact test *P*=0.0003).

We also stained for the CD5L/AIM protein, a soluble 38–40 kDa glycoprotein, also known as Sp*α*, which was expressed by macrophages and eosinophils. The number of positive cells did not vary according to cHL subtype (data not shown).

**Figure 4:**
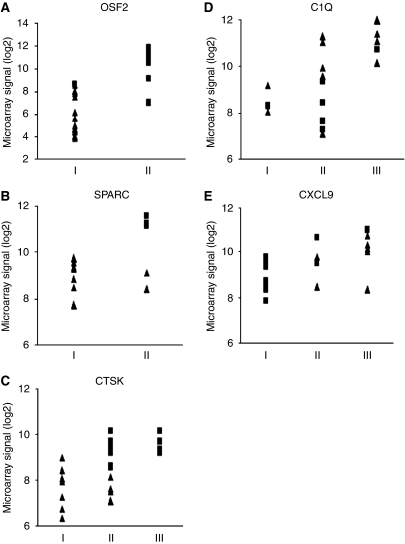
Validation of the microarray results using immunohistochemistry. The tumours were divided according to the staining patterns illustrated in Table 2 and Figure 3. The *y* axis shows the gene expression signal and the *x* axis shows the immunohistochemistry results. NS tumours are denoted by a ▪ and the MC tumours are denoted by a ▴ in the diagrams. (**A**) OSF2 staining, the tumours were divided into two groups. (**B**) SPARC staining, the tumours were divided into two groups. (**C**) CTSK staining, the tumours were divided into three groups. (**D**) C1Q staining, the tumours were divided into three groups. (**E**) CXCL9 staining, the tumours were divided into three groups.

In Figure 4, the correlation between the gene expression signals and the immunohistochemistry results is illustrated. The aim was to identify which cells expressed proteins coded by identified genes. Therefore, the division in various patterns was based not only on the number of cells or the strength of positive staining, but also on which cell types were positive. In some cases, the combined expression of two cell types might have the same expression level as one cell type in other cases.

## Discussion

With our combined approach of gene expression profiling and *in situ* analysis of tumour sections, we have advanced the understanding of molecular differences that define the two major subtypes of cHL. The difference in the gene expression profile between NS and MC at the tissue level is dominated by an apparent mimic of different phases of the normal wound-healing process. These differences relate primarily to the phenotypes of the infiltrating macrophages and fibroblasts. Our data suggest that most macrophages in the NS subtype might display the so-called M2 phenotype because of their ECM remodelling profile, whereas those infiltrating MC tumours are similar to cytotoxic macrophages found in other types of lymphomas (Dave *et al*, 2004; de Jong *et al*, 2009).

Many NS-related genes were associated with extracellular deposition and some of the MC-related genes were associated with cytotoxic T cells and IFN*γ* signalling, also in line with earlier publications (Teruya-Feldstein *et al*, 1999). Our results suggest that these ECM depositions in part mimic the wound-healing processes. Wound healing can be divided into three different phases: the inflammatory phase, the proliferative phase and the remodelling phase. The inflammatory phase is characterised by an increase of stimulated epithelial and endothelial cells producing cytokines and chemokines that recruit and activate neutrophils, macrophages, T cells, B cells and eosinophils. In the subsequent proliferative phase, macrophages are activated leading to degradation of the contemporary ECM, myofibroblasts produce new ECM components (especially collagen III) and endothelial cells form new blood vessels. This is followed by a remodelling and maturation phase when collagen fibres become more organised (collagen I replaces collagen III), blood vessels are normalised and scar tissue is eliminated (Weinberg, 2007). The inflammatory components of the gene expression pattern in MC are similar to those of the inflammatory wound-healing phase, whereas NS resembles the proliferative/tissue remodelling phase.

More than 200 genes showed subtype-specific expression in NS and MC cHL. Most of these genes showed a higher expression in the NS subtype, whereas about 50 had a higher expression in the MC cHL. Several of the NS-related genes were regulated by TGF*β* and/or IL-13. For example, the high expression of *COLI* was specific for NS, whereas *COLIII* was higher in NS than in MC. The promoter of *COLI* contains a TGF*β*-responsive STAT6-binding site (Buttner *et al*, 2004; Bhogal *et al*, 2005).

The differences in expression of subtype-related gene products were seen primarily in macrophages and fibroblasts. However, the level of macrophage infiltration was heterogeneous within both cHL subtypes. In certain tumours, macrophages positive for NS-related genes, such as cathepsin K, were evenly scattered throughout the tissue, whereas in other samples they clustered around ECM deposits. It has been postulated that fibroblasts take over the wound-healing process in an inflammatory environment where TGF*β* is predominant, leading to an excess of fibrosis similar to that seen in the proliferative and tissue remodelling phases of wound healing (Provenzano *et al*, 2005). Tissue growth factor-*β* is also associated with the polarisation of macrophages into the so-called M2 phenotype associated with tissue-remodelling activities (Mantovani *et al*, 2002; Sica *et al*, 2008).

Our results suggest that the NS-related macrophage activity of ECM remodelling is not present in the MC subtype of cHL. This is supported by the increased STAT1 phosphorylation, *CXCL9* expression and the expression of C1Q subunits, which are all controlled by IFN*γ*, a cytokine that has earlier been reported to be increased in the MC subtype (Teruya-Feldstein *et al*, 1999). It has earlier been shown that STAT1 activation in macrophages is linked to prognosis in cHL (Sanchez-Aguilera *et al*, 2006). The level of TAMs has been indicated to have poor prognostic impact on a number of cancers, including breast cancer and follicular lymphomas (Leek *et al*, 1996; Dave *et al*, 2004; Shunyakov *et al*, 2004; Sanchez-Aguilera *et al*, 2006). In a mouse model, it was shown that STAT1 is activated and that the *CXCL9* and C1Q subunits are expressed in TAMs (Biswas *et al*, 2006). The C1Q subunits are membrane-bound molecules that are associated with increased phagocytic activity in macrophages (Porta *et al*, 2007).

The H-RS cells are thought to orchestrate the composition of the tumour tissue by secreting inflammatory proteins and cytokines and thereby controlling the infiltration of reactive cells. The infiltration of non-malignant cells such as eosinophils and mast cells has been reported to predict the outcome in cHL (Axdorph *et al*, 2001; Molin *et al*, 2002). Various reactive cells could contribute to malignancies such as cHL by releasing extracellular proteases, proangiogenic factors and chemokines (Coussens and Werb, 2002; Chiu *et al*, 2007). In view of the lack of an animal or *in vitro* model system for cHL, it is difficult to study whether the microenvironment affects the H-RS cells or vice versa. One could hypothesise that the malignant H-RS cell is responsible for the morphological differences characteristic of the subtypes, and thus the expression of factors produced by the H-RS cells should differ between the subtypes. Some minor subtype-specific differences in the profiles of the H-RS cells have been reported. Phosphorylated STAT6 is more commonly found in the H-RS cells of the NS subtype compared with the MC cHL (Skinnider *et al*, 2002). Expression of certain kinases vary between the H-RS cells in the two subtypes depending on the EBV status (Renne *et al*, 2007). However, the gene expression profiles of the H-RS cells isolated by laser capture showed no significant differences related to cHL subtype or EBV status (Karube *et al*, 2006).

Taken together, inflammatory or wound healing-related programmes, where fibroblasts and macrophages have a dominant function, seem to distinguish between the NS and MC cHL subtypes.

## Figures and Tables

**Table 1:** Summary of the evaluation of fibrosis and number of cells

	**Subtype**	**Fibrosis**	**Eosinophilia**	**Macrophages**	**Cohort number**
1	MC	0	35	Low	SWE4
2	MC	1	2	High	SWE7
3	MC	2	6	High	SWE9
4	MC	1	10	High	SWE31
5	MC	2	19	Low	SWE32
6	MC	0	15	Low	SWE33
7	MC	0	5	High	SWE34
8	MC	2	5	High	SWE35
9	MC	1	31	High	SWE37
10	MC	2	3	High	SWE38
11	MC	1	26	High	SWE41
12	MC	1	74	Low	SWE42
13	MC	0	14	High	SWE43
14	MC	0	5	High	SWE50
15	NS	2	154	Low	SWE44
16	NS	3	108	Low	SWE45
17	NS	1	40	High	SWE46
18	NS	3	142	High	SWE47
19	NS	2	30	High	SWE48
20	NS	2	15	Low	SWE49
21	NS	1	27	High	SWE51
22	NS	3	39	Low	SWE52
23	NS	2	73	High	SWE53
24	NS	1	90	High	SWE55
25	NS	2	214	Low	SWE56
26	NS	3	161	Low	SWE58
27	NS	1	4	High	SWE59
28	NS	2	25	High	SWE60

Abbreviations: MC=mixed cellularity; NS=nodular sclerosis.

**Table 2:** Immunohistochemical patterns of studied gene product expression

*OSF2*	
Group I	Low staining
Group II	Strong positive staining in fibrotic bands and fibroblasts
	
*SPARC*	
Group I	Low number of macrophages positive
Group II	High staining in macrophages and fibroblasts
	
*CTSK*	
Group I	Low staining
Group II	High staining in macrophages and fibroblasts
Group II	Extensively high staining in macrophages, fibroblasts and lymphocytes
	
*C1Q*	
Group I	Positive in lymphocytes, low in macrophages
Group II	Positive in lymphocytes and up to 50% of macrophages
Group III	Positive in lymphocytes and 50–100% or macrophages
	
*CXCL9*	
Group I	No/low number of cells
Group II	Positive in lymphocytes
Group III	Positive in macrophages and lymphocytes

**Table 3:** Selected genes differentially expressed in the NS and MC subtypes of cHL^a^

**Gene symbol**	**Description**	**FC (NS/MC)**	**FDR**
*ECM subunits*			
OSF2	Osteoblast-specific factor	1264 300	0
COL3A1	Collagen, type III, *α*1	782 520	0
COL1A1	Collagen, type I, *α*1	646 430	0
LUM	Lumican	527 060	0
COL1A2	Collagen, type I, *α*2	421 190	0
LAMB1	Laminin, *β*1	332 030	0
			
*ECM remodelling genes*			
SPARC	Secreted protein, acidic, cysteine-rich (osteonectin)	294 540	0
CTGF	Connective tissue growth factor	258 620	0
CTSK	Cathepsin K (pycnodysostosis)	229 730	0
MMD	Monocyte to macrophage differentiation-associated	175 870	2,00E-04
			
*Inflammation-associated genes*			
CCL17	Chemokine (C-C motif) ligand 17	378 390	0
CCL22	Chemokine (C-C motif) ligand 22	248 330	0
			
*Receptors*			
CD5L	CD5 antigen-like (scavenger receptor cysteine-rich family)	−197 472	0
ECGF1	Endothelial cell growth factor 1 (platelet-derived)	−190 512	0
EPOR	Erythropoietin receptor	−188 608	0
			
*IFNg-regulated genes*			
C1QB	Complement component 1, q subcomponent, *β*-polypeptide	−227 169	0
CXCL9	Chemokine (C-X-C motif) ligand 9	−189 251	0
C1QA	Complement component 1, q subcomponent, *α-*polypeptide	−185 839	0
IRF6	Interferon regulatory factor 6	−175 377	0

Abbreviations: cHL=classical Hodgkin's lymphoma; ECM=extracellular matrix; FC=fold change; FDR=false discovery rate; MC=mixed cellularity; NS=nodular sclerosis.

a Full list is provided in Supplementary Table 1.

**Table 4:** Validation of microarray results using RT–PCR

	**Correlation coefficient**	**Microarray FC**	**RT–PCR FC**
SPARC	0.95	2,95	3,76
CCL17	0.82	3,80	4,68
CCL22	0.63	2,48	14,96
C1q	0.61	−2,27	−1,8
CXCL9	0.91	−1,89	−5,68

Abbreviations: FC=fold change; RT–PCR=real-time–PCR.

**Table 5:** Frequency of various immunohistochemical patterns of studied gene products in NS and MC cHL

	**Protein**	**Pattern**		**Protein**	**Pattern**		**Protein**	**Pattern**			**Protein**	**Pattern**			**Protein**	**Pattern**		
	**OSF2, *N*=23**	**I**	**II**	**SPARC, *N*=20**	**I**	**II**	**CTSK, *N*=20**	**I**	**II**	**III**	**C1Q, *N*=28**	**I**	**II**	**III**	**CXL9, *N*=24**	**I**	**II**	**III**
MC	15	15	0	13	9	4	13	8	5	0	17	2	3	11	14	0	4	10
NS	8	1	7	7	0	7	7	0	3	4	11	1	7	1	10	7	2	1

Abbreviations: cHL=classical Hodgkin's lymphoma; MC=mixed cellularity; NS=nodular sclerosis.
