# Supplementary material for: Inflammation and tissue repair markers distinguish the nodular sclerosis and mixed cellularity subtypes of classical Hodgkin's lymphoma
Source: Br J Cancer. 2009 Sep 22;101(8):1393–401. doi: 10.1038/sj.bjc.6605238 (PMC2768440; doi:10.1038/sj.bjc.6605238)
Supplement: Supplementary Table 1 [file 6605238x1.doc]

| Supplementary Table 1. Genes differentially expressed in NS and MC cHL |  |
| --- | --- |
| Name | NS/MC FC |
| collagen, type I, alpha 1 | 10,74 |
| versican | 9,80 |
| periostin, osteoblast specific factor | 9,36 |
| interleukin 9 | 9,20 |
| collagen, type III, alpha 1 (Ehlers-Danlos syndrome type IV) | 6,70 |
| matrix metallopeptidase 1 (interstitial collagenase) | 5,79 |
| collagen, type V, alpha 2 | 5,69 |
| procollagen-lysine, 2-oxoglutarate 5-dioxygenase 2 | 5,65 |
| carbonic anhydrase XII | 5,61 |
| collagen, type I, alpha 2 | 5,04 |
| thrombospondin 2 | 5,01 |
| chemokine (C-C motif) ligand 11 | 4,80 |
| inhibin, beta A | 4,77 |
| cartilage oligomeric matrix protein | 4,72 |
| secreted frizzled-related protein 4 | 4,66 |
| lysyl oxidase | 4,66 |
| collagen, type V, alpha 1 | 4,57 |
| tryptophan 2,3-dioxygenase | 4,40 |
| matrix metallopeptidase 2 | 4,28 |
| insulin-like growth factor 1 (somatomedin C) | 4,20 |
| fibulin 1 | 3,94 |
| six transmembrane epithelial antigen of the prostate 1 | 3,85 |
| tumor necrosis factor, alpha-induced protein 6 | 3,85 |
| platelet-derived growth factor receptor, alpha polypeptide | 3,78 |
| collagen, type XV, alpha 1 | 3,62 |
| v-fos FBJ murine osteosarcoma viral oncogene homolog | 3,61 |
| chemokine (C-X-C motif) ligand 6 (granulocyte chemotactic protein 2) | 3,55 |
| fibroblast activation protein, alpha | 3,43 |
| lumican | 3,35 |
| secreted protein, acidic, cysteine-rich (osteonectin) | 3,34 |
| collagen, type XI, alpha 1 | 3,17 |
| prostaglandin I2 (prostacyclin) synthase | 3,03 |
| lysyl oxidase-like 2 /// ectonucleoside triphosphate diphosphohydrolase 4 | 2,98 |
| EGF-like-domain, multiple 6 | 2,98 |
| platelet-derived growth factor receptor-like | 2,96 |
| collagen, type XVI, alpha 1 | 2,94 |
| nidogen 2 (osteonidogen) | 2,94 |
| laminin, beta 1 | 2,92 |
| cytoskeleton-associated protein 4 | 2,90 |
| fibrillin 1 | 2,90 |
| asporin | 2,80 |
| chemokine (C-C motif) ligand 17 | 2,79 |
| collagen, type IV, alpha 1 | 2,72 |
| connective tissue growth factor | 2,72 |
| prostaglandin-endoperoxide synthase 2 | 2,68 |
| platelet derived growth factor D | 2,66 |
| microfibrillar-associated protein 2 | 2,63 |
| collagen, type VI, alpha 3 | 2,59 |
| serpin peptidase inhibitor, clade E (nexin, plasminogen activator inhibitor type 1) | 2,59 |
| regulator of G-protein signaling 2, 24kDa | 2,55 |
| cathepsin K | 2,52 |
| latexin | 2,51 |
| serpin peptidase inhibitor, (collagen binding protein 1) | 2,49 |
| ribonuclease, RNase A family, 1 (pancreatic) | 2,48 |
| KDEL (Lys-Asp-Glu-Leu) endoplasmic reticulum protein retention receptor 3 | 2,47 |
| procollagen-proline, 2-oxoglutarate 4-dioxygenase (proline 4-hydroxylase) | 2,45 |
| tropomyosin 1 (alpha) | 2,45 |
| AE binding protein 1 | 2,42 |
| retinoic acid receptor responder (tazarotene induced) 2 | 2,40 |
| inhibitor of DNA binding 4, dominant negative helix-loop-helix protein | 2,38 |
| HtrA serine peptidase 1 | 2,35 |
| regulator of G-protein signaling 5 | 2,34 |
| fibulin 2 | 2,33 |
| laminin, alpha 4 | 2,32 |
| dermatopontin | 2,29 |
| glutamine-fructose-6-phosphate transaminase 2 | 2,28 |
| snail homolog 2 (Drosophila) | 2,27 |
| dehydrogenase/reductase (SDR family) member 2 | 2,26 |
| epithelial membrane protein 1 | 2,26 |
| chemokine (C-X-C motif) ligand 1 (melanoma growth stimulating activity, alpha) | 2,23 |
| serpin peptidase inhibitor, clade F | 2,21 |
| osteopontin, bone sialoprotein I, early T-lymphocyte activation 1 | 2,19 |
| tropomyosin 2 (beta) | 2,18 |
| endothelin receptor type A | 2,17 |
| nicotinamide N-methyltransferase | 2,17 |
| trophoblast glycoprotein | 2,16 |
| polo-like kinase 2 (Drosophila) | 2,15 |
| immunoglobulin superfamily containing leucine-rich repeat | 2,14 |
| G0/G1switch 2 | 2,14 |
| amphiphysin | 2,11 |
| latent transforming growth factor beta binding protein 2 | 2,11 |
| flavin containing monooxygenase 1 | 2,11 |
| killer cell lectin-like receptor subfamily B, member 1 | 2,11 |
| tissue factor pathway inhibitor 2 | 2,08 |
| laminin, gamma 1 (formerly LAMB2) | 2,06 |
| dual specificity phosphatase 1 | 2,02 |
| aldehyde dehydrogenase 1 family, member A3 | 2,01 |
| podocalyxin-like | 2,01 |
| epidermal growth factor receptor pathway substrate 8 | 2,01 |
| neuropilin 1 | 2,00 |
| lectin, galactoside-binding, soluble, 1 (galectin 1) | 1,99 |
| interleukin 1 receptor, type I | 1,99 |
| serpin peptidase inhibitor, clade E , member 2 | 1,99 |
| 3'-phosphoadenosine 5'-phosphosulfate synthase 2 | 1,96 |
| junctional adhesion molecule 2 | 1,96 |
| fibronectin leucine rich transmembrane protein 2 | 1,95 |
| podoplanin | 1,93 |
| peptidylprolyl isomerase C (cyclophilin C) | 1,93 |
| LIM and cysteine-rich domains 1 | 1,93 |
| insulin-like growth factor binding protein 4 | 1,92 |
| insulin-like growth factor 2 (somatomedin A) | 1,92 |
| peripheral myelin protein 22 | 1,91 |
| wingless-type MMTV integration site family, member 5A | 1,90 |
| A kinase (PRKA) anchor protein (gravin) 12 | 1,90 |
| solute carrier family 7, (cationic amino acid transporter, y+ system) member 11 | 1,86 |
| plastin 3 (T isoform) | 1,86 |
| WW domain binding protein 5 | 1,86 |
| vanin 1 | 1,86 |
| aldo-keto reductase family 1, member C3 | 1,86 |
| cholesterol 25-hydroxylase | 1,85 |
| cadherin 11, type 2, OB-cadherin (osteoblast) | 1,84 |
| transforming growth factor, beta-induced, 68kDa | 1,83 |
| deleted in liver cancer 1 | 1,81 |
| TIMP metallopeptidase inhibitor 1 | 1,81 |
| sushi-repeat-containing protein, X-linked | 1,80 |
| integrin, alpha V (vitronectin receptor, alpha polypeptide, antigen CD51) | 1,80 |
| Thy-1 cell surface antigen | 1,79 |
| inhibitor of DNA binding 1, dominant negative helix-loop-helix protein | 1,79 |
| PDZ and LIM domain 3 | 1,79 |
| protocadherin 17 | 1,79 |
| phosphodiesterase 1A, calmodulin-dependent | 1,79 |
| procollagen C-endopeptidase enhancer | 1,78 |
| heparan sulfate proteoglycan 2 | 1,78 |
| insulin-like growth factor binding protein 2, 36kDa | 1,78 |
| vitamin D (1,25- dihydroxyvitamin D3) receptor | 1,78 |
| SRY (sex determining region Y)-box 4 | 1,78 |
| membrane metallo-endopeptidase | 1,77 |
| roundabout, axon guidance receptor, homolog 1 (Drosophila) | 1,77 |
| endomucin | 1,77 |
| endothelial PAS domain protein 1 | 1,76 |
| protease, serine, 23 | 1,76 |
| annexin A1 | 1,76 |
| selectin P (granule membrane protein 140kDa, antigen CD62) | 1,76 |
| WW domain containing transcription regulator 1 | 1,76 |
| potassium inwardly-rectifying channel, subfamily J, member 8 | 1,75 |
| lysosomal-associated membrane protein 3 | 1,75 |
| actin, alpha 2, smooth muscle, aorta | 1,75 |
| solute carrier family 2 (facilitated glucose transporter), member 10 | 1,74 |
| preferentially expressed antigen in melanoma | 1,73 |
| interleukin 7 receptor | 1,73 |
| RAR-related orphan receptor A | 1,73 |
| biglycan | 1,73 |
| cartilage intermediate layer protein, nucleotide pyrophosphohydrolase | 1,73 |
| latent transforming growth factor beta binding protein 1 | 1,72 |
| insulin-like growth factor binding protein 3 | 1,71 |
| interleukin 26 | 1,71 |
| tryptase alpha/beta 1 | 1,71 |
| fibulin 5 | 1,71 |
| secretogranin II (chromogranin C) | 1,71 |
| collagen, type XVIII, alpha 1 | 1,71 |
| thrombospondin 4 | 1,70 |
| activating transcription factor 3 | 1,70 |
| chemokine (C-X-C motif) ligand 11 | -5,83 |
| chemokine (C-X-C motif) ligand 9 | -4,04 |
| chemokine (C-X-C motif) ligand 10 | -2,99 |
| Fc fragment of IgG, high affinity Ia, receptor (CD64) | -2,93 |
| complement component 1, q subcomponent, B chain | -2,93 |
| indoleamine-pyrrole 2,3 dioxygenase | -2,90 |
| prolactin | -2,85 |
| guanylate binding protein 1, interferon-inducible, 67kDa | -2,83 |
| sorting nexin 10 | -2,80 |
| interferon, gamma | -2,74 |
| aldehyde dehydrogenase 1 family, member A1 | -2,67 |
| proline-serine-threonine phosphatase interacting protein 2 | -2,60 |
| CD5 molecule-like | -2,51 |
| granzyme K (granzyme 3; tryptase II) | -2,51 |
| granzyme B (granzyme 2, cytotoxic T-lymphocyte-associated serine esterase 1) | -2,49 |
| perforin 1 (pore forming protein) | -2,42 |
| toll-like receptor 8 | -2,32 |
| complement component 1, q subcomponent, A chain | -2,32 |
| GTP cyclohydrolase 1 (dopa-responsive dystonia) | -2,29 |
| C-type lectin domain family 4, member M | -2,25 |
| carboxypeptidase M | -2,23 |
| tryptophanyl-tRNA synthetase | -2,13 |
| natural killer cell group 7 sequence | -2,08 |
| phospholipase A1 member A | -2,06 |
| granzyme A | -2,02 |
| lymphocyte-activation gene 3 | -1,97 |
| potassium inwardly-rectifying channel, subfamily J, member 2 | -1,90 |
| cytotoxic and regulatory T cell molecule | -1,89 |
| cholesteryl ester transfer protein, plasma | -1,88 |
| leukocyte immunoglobulin-like receptor, | -1,87 |
| chemokine (C-C motif) ligand 21 | -1,87 |
| sialic acid binding Ig-like lectin 1, sialoadhesin | -1,86 |
| chemokine (C-C motif) ligand 5 | -1,86 |
| macrophage receptor with collagenous structure | -1,83 |
| CD72 molecule | -1,80 |
| caspase 5, apoptosis-related cysteine peptidase | -1,80 |
| solute carrier family 31 (copper transporters), member 2 | -1,80 |
| apolipoprotein L, 3 | -1,79 |
| splicing factor 3a, subunit 2, 66kDa | -1,78 |
| retinoic acid receptor responder (tazarotene induced) 3 | -1,78 |
| thymidine phosphorylase | -1,77 |
| leukocyte immunoglobulin-like receptor, subfamily A, member 3 | -1,76 |
| killer cell lectin-like receptor subfamily K, member 1 | -1,76 |
| malic enzyme 1, NADP(+)-dependent, cytosolic | -1,75 |
| oligophrenin 1 | -1,75 |
| CD160 molecule | -1,74 |
| serpin peptidase inhibitor, clade G (C1 inhibitor), member 1 | -1,74 |
| interferon regulatory factor 1 | -1,74 |
| CD8a molecule | -1,73 |
| hypothetical protein LOC8681 | -1,72 |
| Fc fragment of IgE, high affinity I, receptor for; gamma polypeptide | -1,71 |
| tumor necrosis factor, alpha-induced protein 2 | -1,70 |
| dehydrogenase/reductase (SDR family) member 9 | -1,70 |
